# The impact of maternal anxiety disorder on mother-infant interaction in the postpartum period

**DOI:** 10.1371/journal.pone.0194763

**Published:** 2018-05-25

**Authors:** Corinna Reck, Alexandra Tietz, Mitho Müller, Kirsten Seibold, Edward Tronick

**Affiliations:** 1 Ludwig-Maximilians University, Department of Psychology, Munich, Germany; 2 Heidelberg University Hospital, General Psychiatry, Heidelberg, Germany; 3 University of Massachusetts, Boston, United States of America; 4 Harvard Medical School, Boston, United States of America; Chiba Daigaku, JAPAN

## Abstract

**Background:**

This study investigated whether postpartum anxiety disorder is associated to altered patterns of infant as well as maternal engagement in a Face-to-Face-Still-Face interaction (FFSF).

**Sampling and methods:**

*n* = 39 women with postpartum DSM-IV anxiety disorder and *n* = 48 healthy mothers were videotaped during a FFSF with their infant (*M* = 4.1 months).

**Results:**

Infants of the clinical group showed significantly less positive engagement during the play episode than infants of controls. This result depended on infant sex: male controls demonstrated more positive interaction than males of anxious mothers. There was no such effect for female infants who engaged significantly less positively during the play episode than males and did not change their positive engagement during the FFSF. These findings imply pronounced interactive positivity and early vulnerability to maternal anxiety symptoms in male infants. Only the infants of the controls showed the still-face effect. They also protested significantly more during the still-face, while the clinical infants’ protest increased significantly during the reunion. Women of both groups did not differ in their interaction. Maternal intrusiveness was associated to infant protest in the course of the FFSF.

**Conclusions:**

Results suggest that mother-infant intervention should consider affect regulation and infant sex-specific characteristics in anxious mother-infant dyads.

## Introduction

Mother-infant interaction has attracted a vast amount of research attention [1,2,3]. The early interaction between mother and infant is of incredible importance as it creates a stimulating social learning environment for the infant [4,5,6]. It also promotes infant affect regulation while fostering the emotional mother-infant bond [7,8,9,10]. In mother-infant interaction, the infant starts to internalize the implicit rules of social interaction such as mutuality, reciprocity and contingency [11,12,13,14,15,16].

Because of the apparent significance of mother-infant interaction, studies have dealt with the question if maternal mental health problems bear a risk for infant interactive skills [17,18,19,20,21]. In light of the high prevalence rates of up to 13% for postpartum depression [22,23,24, 25] and 12% for postpartum anxiety disorders [26,27,28,23], research in this field is clearly needed. Postpartum depressive disorders have been linked to poorer infant cognitive performance and behavioural problems [29,30,31,32] as well as emotional self-regulation difficulties during mother-infant interaction (e.g. increased infant negative affect) [11,19,33,34]. These adverse developmental outcomes have been linked to lower interactive responsiveness, sensitivity and contingency in depressed mothers [35,36,37,38,39].

Studies on infant interaction in case of maternal postpartum anxiety disorder are sparse. Some pointed out higher stress reactivity during free play in infants of mothers with anxiety disorder compared to infants of controls [18,40]. Moreover, infants of mothers with anxiety disorder were less socially engaged than healthy infants, reflected in less alertness, social initiation, vocalisations, gaze maintenance and positive affect [18]. Infants of socially phobic mothers were also less socially responsive with strangers than infants of controls [41]. Besides, low maternal trait anxiety went along with increased infant explorative play at three to nine months [42].

As the majority of research has dealt with the interactive behaviour of anxious mothers, a greater number of results is available on maternal interaction; yet, the outcomes are inconsistent. Several findings suggest that severely anxious mothers interact less sensitively with their infant than controls [42,18,43,40], while others could not find deficits in this regard [41]. In addition, women with pronounced anxiety exhibited over-aroused / fearful interaction behaviour, leading to vigilance and in some modalities to withdrawn behaviour [44]. Other research has linked anxious parenting to higher parental control [45,46,47] (e.g. excessive regulation of child behaviour, high intrusiveness). In line with these findings, mothers with a DSM-IV anxiety disorder [48] were less granting of autonomy with their ten year old child than healthy mothers [49]. Anxious mothers were also less warm and positive as well as more critical and catastrophising than their healthy counterparts. Unfortunately, research with younger children is limited.

Research revealed certain risk factors that may act in concert with maternal psychopathology in shaping infant developmental outcomes. Of special interest has been infant sex. For example, depressed mother-son dyads experienced more negative affective states and greater difficulties in resuming interaction after an artificially induced interruption than other dyads [50]. Moreover, male infants struggled more with maintaining affective regulation in challenging mother-infant interaction [51] and were more socially oriented [51] than females. Male infants of depressed mothers were also more vulnerable to exhibit impaired cognitive abilities compared to female infants [30,52,53]. Altogether, male infants might be more susceptible to detrimental effects of maternal mental health problems than female infants or more susceptible to environmental influences in general [54].

While most of the mentioned research involved free-play or standardized play, some authors chose the “Face-to-Face-Still-Face” paradigm (FFSF) [55,56] to investigate mother-infant interaction. This procedure assesses how mother-infant dyads manage emotionally stressful situations with the “still-face episode”, a sudden interruption of interaction by the mother which evokes a prolonged state of emotional and behavioural mismatch (uncoordinated behavioural and affective states) [39]. Such maternal disengagement represents an interactive stressor to the infant [57], causing considerable alterations of the infant’s affective and self-regulatory behaviour (“still-face effect”) [58,59,60,61]. The transition from the still-face to the reunion episode illustrates the mutual regulatory skills of the mother-infant dyad [56].

Little is known about the interactive behaviour of mother-infant dyads in a regulatory task such as the still-face with regard to postpartum anxiety disorder. By means of the FFSF paradigm, Grant and colleagues [62] demonstrated that maternal sensitivity moderated the influence of prepartum anxiety disorder on seven-months-old infants’ responses to the still-face. More precisely, lower maternal sensitivity was accompanied by higher infant distress and negative affect during the still-face episode. Thus, it may not be the anxiety disorder itself that negatively impacts infant interaction but a combination of maternal anxiety and low sensitivity. This idea is supported by findings of our research group showing that maternal positive behaviour and dyadic regulatory processes in mother-infant interaction moderated the relationship between maternal anxiety, infant regulatory problems and infant distress [14,9,63]. In another study using the FFSF procedure, infants of mothers with DSM-IV anxiety disorder were actually less likely to express negative affect in the still-face and stranger challenge than infants of controls [64]. The authors argue that the reduced infant negative affect might reflect a distinct coping style of anxious mother-infant dyads in socially challenging situations.

The heterogeneous outcomes with regard to maternal anxiety and mother-infant interaction may be due to several aspects. First, different methods were used to assess interaction, e.g. free play, FFSF procedure. Second, whereas some researchers measured the degree of anxiety with dimensional self-report questionnaires, others relied on a DSM-IV diagnosis. Third, the majority of studies stressing differences between anxious and non-anxious mother-infant dyads, included women with comorbid depression. Although this reflects the high comorbidity of both disorders [65,23,66], it remains unclear which symptomatology caused the inter-group differences.

Because of the mixed findings in this field, clarification is needed whether specific infant interaction patterns are indeed associated to maternal anxiety disorder. Given the high frequency of postpartum anxiety disorders and their potential impact on mother-infant interaction, research in this area is clearly indicated. Therefore, after having conducted a variety of studies dealing with postpartum depression [39,67,68,20], our research group shifted their focus from depressive disorder to anxiety disorders in the postpartum period [69,70]. With regard to the mother-infant relationship, attention was first paid to the question if maternal bonding is impacted by maternal anxiety disorder in the postpartum period [71]. Now it is of interest whether the interaction of anxious mother-infant dyads differs from the interaction of healthy dyads. This is one of the few studies to our knowledge comprising women with anxiety disorders according to DSM-IV without other current psychological disorders. In many studies comorbid depression was not sufficiently controlled, making it difficult to draw conclusions about anxiety disorders.

The main goal of this prospective study was to examine the relationship between DSM-IV postpartum anxiety disorder and infant interaction as most studies have focused on maternal interactive behaviour so far. The limited empirical data about postpartum anxiety disorder and infant interaction predominantly pointed out less positive and more negative affect. Our confirmatory hypotheses are therefore that infants of mothers with anxiety disorder show less positive and more negative interactive behaviour in the FFSF than infants of controls. Because of the heterogeneous outcomes reported for maternal interactive behaviour, the interaction of clinical and control mothers is compared in additional exploratory analyses [72]. As the interactive and parenting behaviour of anxious women has been described as controlling and intrusive [45,47,46,49] which may be reflected in infant negative interaction behaviour [36], we also tested exploratory whether maternal intrusiveness and infant negative engagement (protest) are related. Due to the lack of empirical data on infant social monitoring and object engagement in the context of maternal anxiety disorders, we also tested the remaining infant engagement phases (see S1 File) in exploratory analyses. Since empirical data suggests interactive behaviour to be a function of infant sex, indicating more difficulties in mother-son dyads, we investigated infant sex as an additional factor in all analyses.

## Method

### Participants

The sample stems from a comprehensive longitudinal study dealing with postpartum anxiety disorder, mother-infant interaction and infant development [73,71]. The presented data refer to the first assessment at an average infant age of four months (mother-infant interaction and diagnostic assessment). Subjects were recruited between June 2006 and October 2010 in a German town by means of flyers, newspaper advertisements and public birth announcements. Women were also recruited from the General Psychiatry and the University Women’s Hospital. Initially, the sample comprised *N* = 122 women. Study approval was obtained from the Ethics Committee of the Medical Faculty of Heidelberg University. Written informed consent was obtained and capacity to consent was determined by the clinical psychologists who carried out the clinical interview at the first assessment.

**Fig 1:**
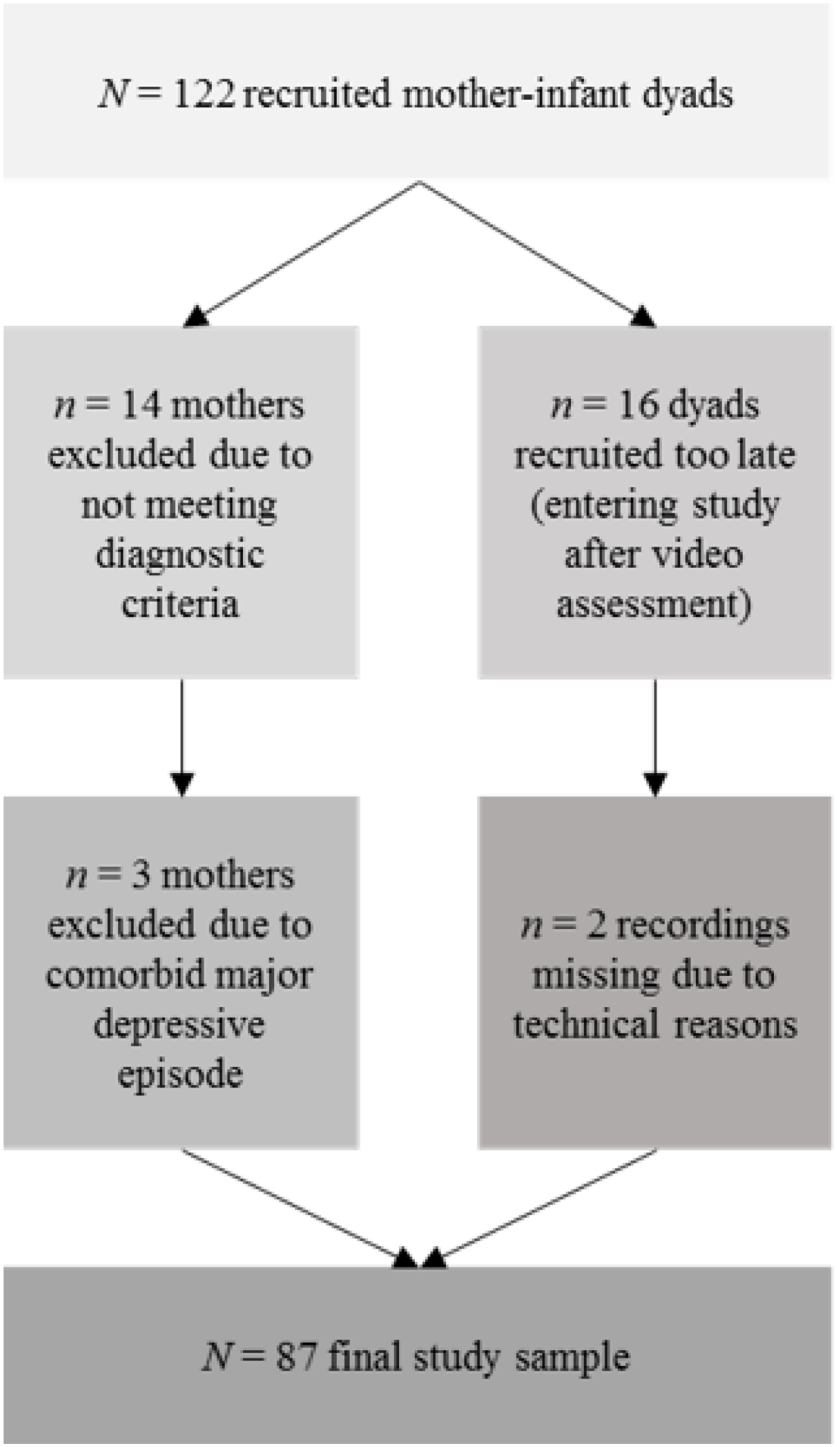
Flow chart with case exclusions and missing data.

A full version of the German Structured Clinical Interview for DSM-IV Disorders (SCID-I) [74] was carried out for a classification as a healthy or anxious mother. Women of the clinical sample had to fulfil the criteria for a DSM-IV diagnosis of anxiety disorder [75]. Women with a lifetime diagnosis of psychosis and bipolar disorder were not included. Despite initial screening efforts to exclude mothers with any comorbid psychological disorder, *n* = 3 women of the clinical group were diagnosed with comorbid major depression at the first assessment. These cases were excluded because they showed different interaction than the clinical sample on a descriptive level (e.g. pronounced intrusiveness). Healthy controls should neither have a current nor lifetime SCID-I diagnosis nor have received psychotherapy at any time of their life. A total of N = 122 women was approached. *n* = 14 women did not meet diagnostic criteria after the first assessment (including the *n* = 3 mothers with comorbid depression) and further *n* = 16 women were recruited at a later study entry (after the video assessment). Consequently, *N* = 89 dyads were assessed for the relevant study variables. Of this sample the video recordings of *n* = 2 dyads were missing due to technical reasons (compare Fig 1). All infants were healthy and had a gestational age at birth of no less than 37 weeks.

To assure that this procedure of list-wise case-exclusions was valid for our data set, we used Little’s MCAR-test [76]. This test evaluates if the missing-completely-at-random-condition (MCAR) is fulfilled. If not significant, differences between the excluded cases and the remaining sample are unlikely. For the MCAR-test, we considered the following variables: maternal age and education, marital status, number of children, infant age and sex, birth mode, gestation age, APGAR score, diagnostic group and interaction data (ICEP-R). The test turned out to be non-significant (χ^2^ = 134.001, *df* = 218, *p* > .999), i.e. list-wise case-exclusions are valid for our sample and the sub-population is representative for the larger sample. The final sample (*N* = 87) is comprised of *n* = 39 Caucasian women with postpartum anxiety disorder and *n* = 48 Caucasian healthy women without a history of mental health disorders, each with their infant. In the clinical sample, *n* = 24 women suffered from more than one DSM-IV anxiety disorder. *n* = 21 women had a generalized anxiety disorder. *n* = 17 women were diagnosed with panic disorder with or without agoraphobia or agoraphobia without history of panic disorder. *n* = 17 women had an obsessive-compulsive disorder. *n* = 9 women were diagnosed with social phobia, while *n* = 1 woman suffered from post-traumatic stress disorder and *n* = 4 women were diagnosed with an anxiety disorder not otherwise specified. According to a retrospective self-report of the participants, *n* = 7 women had a postpartum onset of anxiety disorder. All women were symptomatic at the time of assessment.

Within the overall sample, the age of women ranged from 22 to 45 years with a mean age of *M* = 33.0 years (*SD* = 5.6 years). Infants had a mean age of *M* = 4.1 months (*SD* = 1.5 months). With regard to the total sample, *n* = 54 (62.1%) of the infants were female. The distribution of maternal education in the overall sample was as follows: four mothers (4.6%) had low level secondary education, *n* = 16 (18.4%) completed intermediate secondary education, *n* = 19 (21.8%) qualified for university entrance and *n* = 48 (55.2%) of the mothers held a university degree. More than half of the sample had one child including the index infant (*n* = 54, 62.1%). *n* = 26 mothers (29.9%) had two children. *n* = 7 (8.0%) study infants had two or more siblings. Two thirds of the sample was married (*n* = 58, 66.7%). Overall, the sample is comparable to a representative study sample for this particular region in Southern Germany [23].

### Instruments

#### Assessment of maternal postpartum anxiety

Postpartum anxiety disorder was measured with the German version of the SCID-I [74] by trained interviewers. Based on DSM-IV criteria [75], the following anxiety disorders were considered: panic disorder, panic disorder with agoraphobia, agoraphobia without history of panic disorder, generalized anxiety disorder, social phobia, compulsive-obsessive disorder, post-traumatic stress disorder and anxiety disorder not otherwise specified. Specific phobias were diagnosed; yet, a mere diagnosis of specific phobia was not sufficient to be classified as a clinical subject due to its relatively low clinical relevance for everyday functioning. The SCID-I was also used to check for comorbid or history of other psychological disorders.

#### Assessment of mother-infant interaction

Mother-infant interaction was investigated with Tronick’s FFSF paradigm [55] in a video laboratory. The FFSF is a standardized procedure to analyse infants’ reactions to a socio-emotionally challenging situation. This paradigm also allows comparing baseline play interaction patterns with interaction behaviour after a socio-emotional stressor [77]. To date, the FFSF has been used in many different variations [78]. The procedure used in the presented research study (for a detailed description, please refer to [14]) was as follows: The child was secured in an infant seat vis-à-vis to his/her mother. The FFSF paradigm consisted of three episodes, each of two minutes duration, namely the play, the still-face and the reunion episode. During the initial play episode the mother was instructed to play with her infant as she always does but without the aid of toys or pacifier. After two minutes, a tap-signal from the adjoining room prompted the mother to engage in a transition interval between the play and the following still-face episode. During this interval, the mother turned her head aside while counting quietly to ten. Meta-analytic results show no significant differences in infant gaze or display of positive and negative affect between FFSF procedures with and without transition intervals [78]. After the transition interval, the mother was instructed to look across her infant without engaging in any mimics, gestures or vocalisations (“still-face”). This maternal unresponsiveness creates a prolonged state of interactive mismatch. Two minutes later, a tap-signal prompted the beginning of the concluding reunion episode, in which the mother resumes the face-to-face play with her infant without a transition interval.

The interactive behaviour of infant and mother during the FFSF was coded using the German translation and revision of the micro-analytical Infant and Caregiver Engagement Phases (ICEP)—Heidelberg Version (ICEP-R; see S1 File). The ICEP-R phases are coded mutually exclusive on a frame-by-frame basis combining information of the face, direction of gaze and vocalisations of the infant and caregiver. The ICEP-R engagement phases for the infant are negative engagement (further divided into withdrawn and protest), object/environment engagement, social monitor, and social positive engagement. The ICEP-R codes for the caregiver are negative engagement (further divided into withdrawn, hostile and intrusive), non-infant focused engagement, social monitor/no vocalisations or neutral vocalisations, social monitor/positive vocalisations, and social positive engagement (overview, see S1 File).

Two independent trained raters, who were blind to the study hypotheses and maternal psychiatric status, scored all mother-infant interactions. Systematic coding discrepancies between the coder and trainer were discussed. Coders received re-training for the specific discrepant categories until an inter-rater reliability of at least Cohen’s κ = .70. Due to the mutual exclusiveness of the nominal ICEP-R categories and in line with other studies [79,39,80,50], we used Cohen’s κ [81] to compute inter-rater reliability for maternal and infant codes. In contrast to intra-class correlations (ICCs), κ-coefficients reflect the concordance between coders more suitably as the assumption of exchangeable observers for ICCs may be violated. 32.2% (*n* = 28 dyads) were randomly selected and coded by two independent study coders. Inter-rater reliability was κ = .73 for infant codes and κ = .78 for maternal codes, which is similar to previously reported reliabilities [39,80,51] and can be evaluated as substantial.

### Data analyses

The minimal anonymized dataset (see S2 File) and the dataset description (see S3 File) can be taken from the Supporting Information Files.

#### Dependent measures

The dependent measures, the relative time duration of infant and caregiver interactive behaviours, was calculated as the sum of seconds in which infants and caregiver engage in each ICEP-R category divided by the time of the FFSF episode. For descriptive results, relative time durations are multiplied by 100%. Maternal non-infant focused engagement, withdrawal, hostility and infant withdrawal occurred too rarely (in mean below 1.0% in all phases) to be included in the analyses. We concentrated on infant protest regarding infant negative affect. All mothers maintained a still-face during the still-face episode. None of the video-assessments had to be interrupted or cut short due to instructional failure, feeding, fussiness or the infant falling asleep.

#### Statistical tests

We used the *Statistical Package for Social Sciences* (IBM SPSS v. 24.0.0.0) for all analyses. Power analyses were computed using *G-Power* v. 3.1.9.2 [82,83]. Effects of group, infant sex, FFSF episode and interaction terms were tested by three-way ANOVAs for repeated measures. The two confirmatory hypotheses refer to the main effects of group in a set of two ANOVAs (one with infant positive engagement and one with infant protest behaviour as the outcome). The critical α-errors of the two confirmative analyses were Holm-Bonferroni-adjusted [84]. This sequential procedure controls the family-wise error-rate by adjusting the critical α-level for each of the individual hypotheses. Thus, it is more powerful than a simple Bonferroni-adjustment and it keeps the family-wise error-rate equal or lower than α. By applying the Holm-Bonferroni-procedure, the critical α is set to .025 for the first and .05 for the second ANOVA. The α-errors of the exploratory analyses [72] were not adjusted. As the global α-error cumulates to α_global_ = 1 –(1–0.05)^8^ = .337 for this set of analyses, its results are descriptive and must be interpreted with caution. Trends are neither interpreted for confirmatory nor exploratory analyses. Effect sizes are reported as partial η^2^, which is a sample-based estimator of explained variance. According to Cohen [85], effects sizes of η^2^ = .01 are small, η^2^ = .06 are medium-sized and η^2^ = .14 are large. Mauchly’s procedure was used to test for violation of the assumption of sphericity. If significant, repeated measures *df*s were Huynh-Feldt corrected. This was the case for infant positive engagement (*p* < .01; ε = .918) and infant protest (*p* < .01, ε = .877). As the sample size of the study was reduced by case-exclusions and missing values (see above and Fig 1), the a priori power analysis is not reported. The post hoc power analysis [83] for all ANOVA-effects tested in the current analyses are demonstrated in Table 1. In case of non-significant effects, it is considered that the H_0_ holds if 1-β is equal to or exceeds .80. Thus, regarding between-subject effects, our analyses ran out of power to exclude medium-sized and small effect sizes; regarding within-subject and within-between interaction effects, power was too low to exclude small effect sizes. Dunn’s multiple comparison procedure [86] was used as post-hoc test because it allows hypothesis-driven and economic multiple testing. This procedure results in a minimum significant difference (ψ). The test value is *t*-distributed. Before carrying out the main analyses, differences concerning maternal age, infant age, number of children, maternal education and marital status between controls and their clinical counterparts as well as between male and female infants were explored (via *t*-tests, Mann-Whitney-*U*-tests and χ^2^-tests) to ensure comparability between the groups.

**Table 1:** Post hoc power analysis for defined effect sizes(f).

	effect	between-subject effect	within-subject effect	within-between interaction
ANOVA infants^a^	large^c^	97.5%	100.0%	100.0%
	medium-sized^d^	64.1%	99.9%	99.4%
	small^e^	13.4%	50.0%	32.8%
ANOVA mothers^b^	large^c^	94.3%	100.0%	100.0%
	medium-sized^d^	55.4%	99.9%	99.5%
	small^e^	11.8%	54.0%	37.1%

^a^α = .05, *r* = .05, ε = .95, 3 measures.

^b^α = .05, *r* = .06, ε = 1, 2 measures.

^c^*f* = .40.

^d^*f* = .25

^e^*f* = .10

## Results

### Preliminary data analyses

There was no effect of group or infant sex on maternal age, infant age, maternal education or marital status. Moreover, sex ratio did not differ between infants of the clinical and the control group. Only number of children differed significantly with clinical mothers having fewer children than controls. Descriptive data of subgroups and tests on comparability are presented in Table 2.

**Table 2:** Descriptives of subgroups and tests on comparability.

	control group	clinical group	female infants	male infants
*maternal age (years)* *M* (*SD*)	33.5 (5.6)	32.3 (5.6)	33.1 (5.4)	32.8 (6.0)
*t* (*p*)	0.98	(.33)	0.18	(.86)
*infant age (months)* *M* (*SD*)	3.9 (1.4)	4.4 (1.5)	4.3 (1.6)	3.9 (1.3)
*t* (*p*)	1.76	(.08)	1.21	(.23)
maternal education	control group (*f*)	clinical group (*f*)	female infants (*f*)	male infants (*f*)
university degree	28	20	30	18
university entrance qualification	11	8	10	9
high secondary qualification	8	8	12	4
low secondary qualification	1	3	2	2
*U* (*p*)	837.5	(.35)	875.0	(.88)
one child	24	30	38	16
two children	18	8	12	14
three or more children	6	1	4	3
*U* (*p*)	668.5	(< .01)	707.5	(.06)
not married	10	14	15	9
married	35	23	36	22
χ^2^ (*p*)	2.39^a^	(.12)	< .01^b^	(.97)
female infants	31	23	/	/
male infants	17	16	/	/
χ^2^ (*p*)	0.29^c^	(.59)	/	/

^a^0 cells have expected count of less than 5, minimum expected count is 10.83.

^b^0 cells have expected count of less than 5, minimum expected count is 9.07.

^c^0 cells have expected count of less than 5, minimum expected count is 14.79.

Because of the infant age range of our sample and because number of children may influence maternal caregiving behaviour [87], we checked whether these variables are associated to maternal and infant interaction to detect potential covariates for our main analysis. Number of children was associated to maternal intrusive engagement (play: *r* = -.24, *p* = .02), infant social monitoring (play: *r* = .22, *p* = .04; reunion: *r* = .30, *p* < .01) and infant object engagement (play: *r* = -.23, *p* = .03; reunion: *r* = -.30, *p*< .01). Infant age was significantly associated to infant social monitoring (still-face: *r* = -.31, *p* < .01; reunion: *r* = -.28, *p* < .01), infant object engagement (still-face: *r* = .34, *p* < .01) and marginally to infant positive engagement during the reunion episode (*r* = .188, *p* = .08). Respective ANOVAs were adjusted for infant age and number of children.

### Confirmatory analyses

#### Infant positive engagement

**Fig 2:**
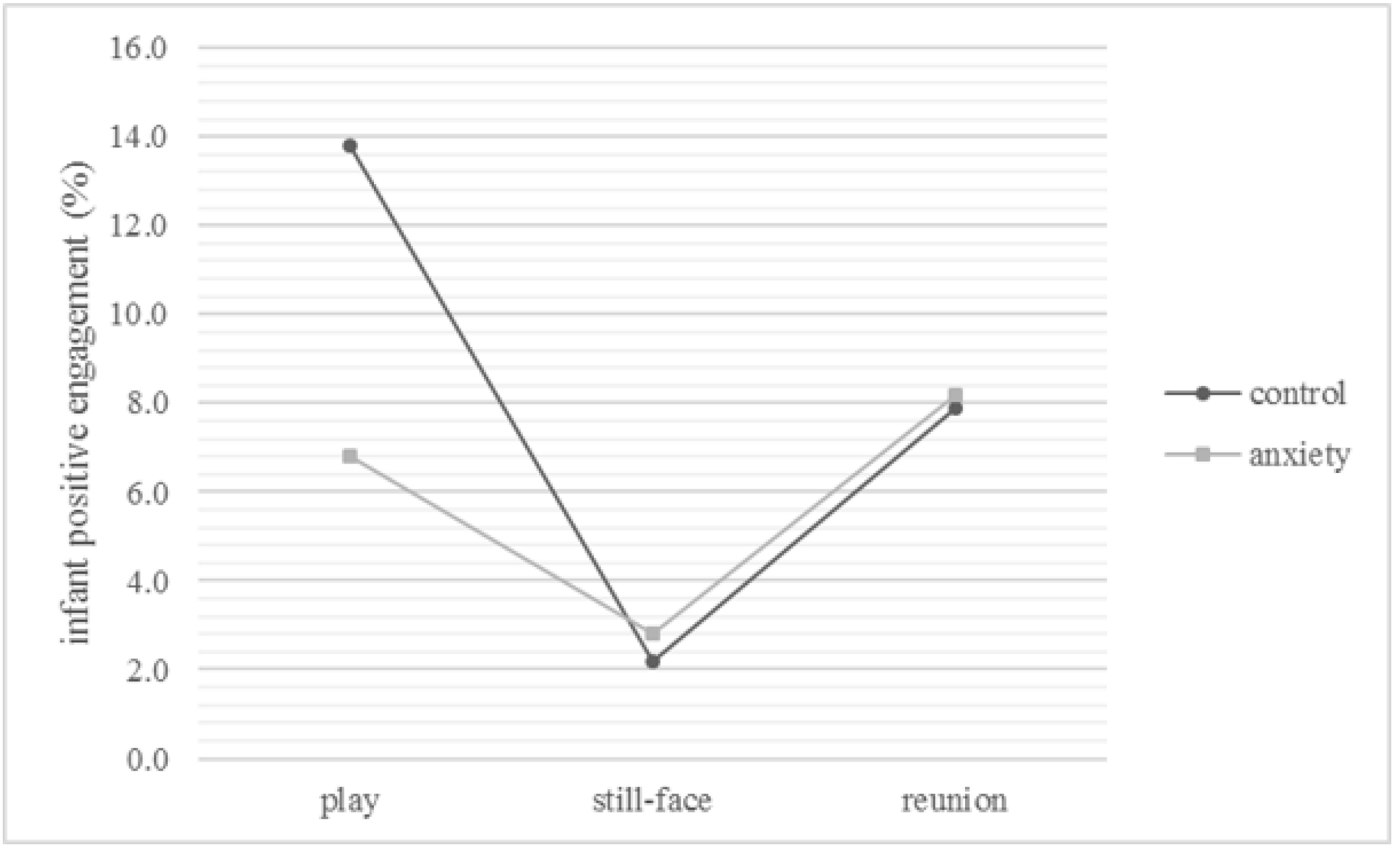
Group x episode interaction of infant positive engagement. The Figure depicts the means in numbers and the standard errors in error bars. Covariates appearing in the model are evaluated at infant age = 4.113.

No significant main effects of group (*F*_1, 82_ = 1.55, *p* = .22), infant sex (*F*_1, 82_ = 3.13, *p* = .08) or episode (*F*_1.8, 150.5_ = 2.65, *p* = .08) were found. The interaction term group x episode was significant (*F*_1.8, 150.5_ = 5.78, *p* < .01, η^2^ = .066), indicating a significant decrease of infant positive engagement from the play to the still-face episode in the control group (see Table 3 and Fig 2). Post-hoc tests (*t* = 2.58, ψ = 4.3%, α = .05) showed that the level of positive engagement in the play and the still-face episode did not differ but increased from the still-face to the reunion episode in the infants of the clinical group. The infants of the control women showed a different pattern: positive engagement of the control infants was higher in the reunion episode than in the still-face but lower than in the play phase. Dunn’s critical difference (ψ = 4.3%) was also exceeded in the comparison of infants of controls with infants of the clinical group during the FFSF play episode: hence, infants of the clinical group demonstrated significantly less positive engagement during the play episode than infants of the controls which is partially consistent with our hypotheses (see Table 3 and Fig 2).

**Table 3:** Means and standard errors of significant interaction effects in infant positive engagement (%)^a^.

group x episode interaction effect		control group			clinical group		
		play	still-face	reunion	play	still-face	reunion
	*M* (*S*.*E*.)	13.8 (1.9)	2.2 (0.8)	7.9 (1.6)	6.8 (2.0)	2.8 (0.8)	8.2 (1.7)
infant sex x episode interaction effect		female infants			male infants		
		play	still-face	reunion	play	still-face	reunion
	*M* (*S*.*E*.)	6.9 (1.7)	2.7 (0.7)	6.9 (1.4)	13.7 (2.1)	2.3 (0.9)	9.2 (1.8)
group x episode x infant sex interaction effect		control group—female infants			clinical group—female infants		
		play	still-face	reunion	play	still-face	reunion
	*M* (*S*.*E*.)	8.1 (2.2)	2.9 (0.9)	8.9 (1.8)	5.7 (2.6)	2.5 (1.1)	4.9 (2.2)
		control group—male infants			clinical group—male infants		
		play	still-face	reunion	play	still-face	reunion
	*M* (*S*.*E*.)	19.4 (3.0)	1.5 (1.2)	6.9 (2.5)	8.0 (3.0)	3.0 (1.3)	11.5 (2.6)

^a^Covariates appearing in the model are evaluated at infant age = 4.113.

Moreover, a significant episode x infant sex interaction (*F*_1.84,150.47_ = 4.33, *p* = .02, η^2^ = .050) was found. Thus, male infants showed more positive engagement during the play than the still-face episode (for means and standard errors see Table 3). Moreover, post-hoc tests (*t* = 2.68, ψ = 4.5%, α = .05) revealed that male infants’ positive engagement increased from the still-face to the reunion episode, while remaining below the level exhibited in the play episode. Furthermore, male infants demonstrated significantly more positive engagement during the play phase than females. The latter showed no significant alteration in positive engagement during the course of the FFSF.

The significant two-way interaction terms were enriched by a significant three-way interaction between group, episode and infant sex (*F*_1.84,150.47_ = 6.521, *p* < .01, η^2^ = .074), indicating a decrease in positive engagement for male control infants from the play to the still-face episode (for means and standard errors see Table 3). Post-hoc tests (*t* = 2.81, ψ = 6.7%, α = .05) suggested further that control males did not exhibit a significant increase of positive engagement in the reunion episode. The positive engagement shown by the male infants of the clinical group followed a different pattern: positive engagement increased significantly from the still-face to the reunion episode. In addition, control males showed higher rates of positive engagement during the play episode than males of clinical mothers. The female infants did not show any changes or group differences over the course of the FFSF. This resembles the before described pattern of the two-way interaction between group and episode.

#### Infant protest

**Fig 3:**
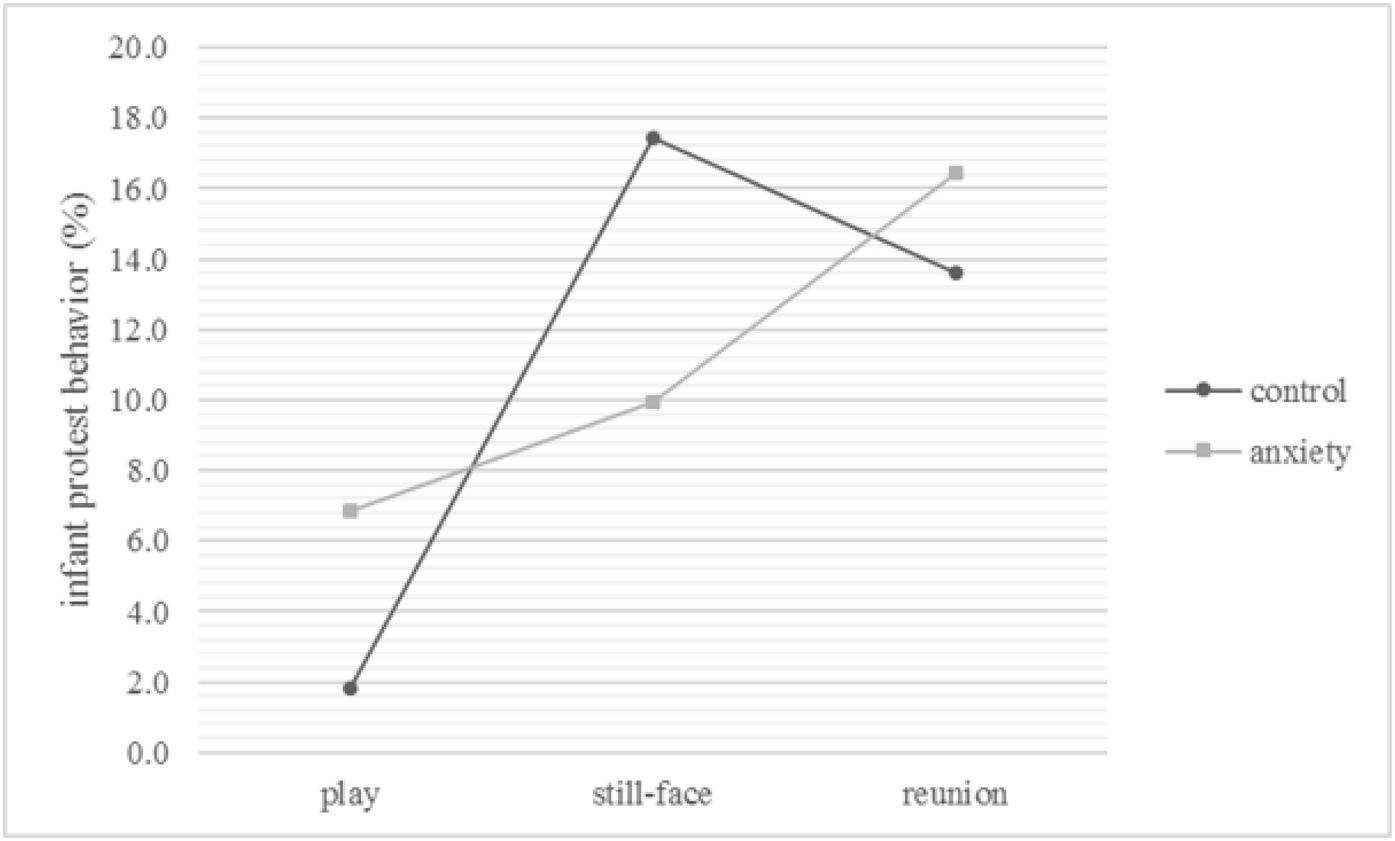
Group x episode interaction on infant protest behavior. The Figure depicts the means in numbers and the standard errors in error bars.

There were no significant main effects of group (*F*_1,83_ < 0.01, *p* = .98) or infant sex (*F*_1,83_ = 2.86, *p* = .10) on infant protest. But the main effect of episode was significant (*F*_1.75,145.53_ = 10.21, *p* < .001; η^2^ = .110), indicating that protest was lowest in the play (*M* = 4.3%, *S*.*E*. = 1.3%) and highest in the reunion episode (*M* = 15.0%, *S*.*E*. = 3.2%) regardless of the group. Post-hoc tests (*t* = 2.24, ψ = 6.0%, α = .05) also revealed that infant protest was lower during the play than the still-face episode (*M* = 13.7%, *S*.*E*. = 2.9%). The effect of episode was enriched by a significant group x episode interaction (*F*_1.75,145.53_ = 3.34, *p* < .05, η^2^ = .039), indicating an increase for control infants from the play (*M* = 1.8%, *S*.*E*. = 1.7%) to the still-face episode (*M* = 17.4%, *S*.*E*. = 4.0%) (Fig 3). Post-hoc tests (*t* = 2.74, ψ = 9.7%, α = .05) suggested that the level of protest shown by the control group did not significantly decrease in the reunion episode (*M* = 13.6%, *S*.*E*. = 4.3%). Infants of mothers with anxiety disorders showed a significant difference between the play (*M* = 6.8%, *S*.*E*. = 1.9%) and the reunion episode (still-face episode: *M* = 9.9%, *S*.*E*. = 4.3%).

### Exploratory analyses

The results of the exploratory analyses [72] are descriptive due to cumulation of α-errors (α_global_ = .337) and aim to generate hypotheses. Since we intended to explore differences between the clinical and the control group, we only report results regarding the main effects of maternal diagnosis and their interaction terms.

#### Infant social monitoring and infant object engagement

There was neither a main effect of diagnosis (infant social monitoring: *F*_1,81_ = 0.22, *p* = .64; infant object engagement: *F*_1,81_ = 0.19, *p* = .67), nor was there any significant interaction term (*p* > .05).

#### Maternal positive engagement and positive vocalization

No main effects of group (maternal positive engagement: *F*_1,83_ < 0.01, *p* = .97; maternal positive vocalisation: *F*_1,83_ = 0.15, *p* = .70) or interaction terms were revealed as significant (*p* > .13).

#### Maternal social monitoring and maternal intrusive engagement

No main effects of group were found (maternal social monitoring: *F*_1,83_ = 0.55, *p* = .46; maternal intrusive engagement: *F*_1,82_ = 0.23, *p* = .63). No interaction terms reached statistical significance (*p* > .16).

#### Correlative findings

Further, we were interested whether there were any associations between maternal intrusive engagement and infant protest behaviour throughout the FFSF. This was the case during the play episode (*r* = .27, *p* = .01). Moreover, infant protest behaviour during the still-face episode was associated to maternal intrusive engagement during the reunion episode (*r* = .23; *p* = .03).

## Discussion

### Summary

This study examined whether anxiety disorders in the postpartum period are associated to altered interaction patterns in infants and their mothers in a FFSF procedure. Although, no main effect of group (clinical versus control) was found, the presented data partially support the hypothesis that postpartum anxiety disorder is associated to less positive and more negative patterns of infant engagement in the challenging FFSF. According to initial results, infants of women with anxiety disorder started with less positive engagement into the FFSF than infants of the controls. Significant interaction effects between group, FFSF episode and infant sex revealed that this group difference was related to infant sex and context. More precisely, male controls demonstrated more positive interaction than male infants of anxious women in the play episode. Moreover, only the control infants demonstrated the typical still-face effect by responding promptly with a decrease in positive engagement and marked protest to maternal withdrawal. Infants of mothers with anxiety disorder, in contrast, exhibited a significant increase in infant protest only between the play and the reunion episode. Both groups recovered in terms of positive engagement during the reunion episode. Women of both groups did not differ significantly in interaction behaviour but maternal intrusiveness correlated positively with infant protest in the course of the FFSF procedure.

### Infant positive interaction behaviour

#### Effects of maternal anxiety disorder

One of the major questions was whether infants of the clinical group show significantly different interaction during the FFSF than control infants. No main effect of maternal anxiety disorder was found, yet, there was a two-way interaction between anxiety disorder and FFSF episode. Post-hoc tests revealed that compared to the controls, the clinical infants initiated with lower rates of positive engagement. They also did not significantly change their positive engagement from the play to the still-face episode; however, their positive engagement increased from the still-face to the reunion episode. In our sample, only the control infants demonstrated the typical still-face effect characterised by less positive engagement and more negative affect (infant protest) from the play to the still-face episode. Moreover, the control infants’ positive affect increased in the reunion episode, yet it remained below the level exhibited in the initial FFSF phase (carry-over effect). It seems that the infants of the healthy women responded more sensitively to the maternal disengagement than the clinical infants; the latter did not show a significant alteration in positive engagement during the interrupted interaction. As there were no group differences in maternal engagement, the question remains why infants of mothers with anxiety disorder might be less sensitive to the presence (play episode) and absence (still-face) of maternal positive effect. Such engagement style may not merely be considered as a weakness or vulnerability in terms of impaired social engagement [18], withdrawal [88] or lowered sensitivity to positive affect. The reduced sensitivity to the play and still-face episode in infants of mothers with anxiety disorder could also be viewed as a strength, provided that differences in maternal interaction exist in case of maternal anxiety [42,18,43,40], which we were not able to detect in the current study. In case of different maternal interaction patterns, the reduced emotional sensitivity might protect infants of mothers with an anxiety disorder from negative interactive experiences with their caregiver. This notion is based on Belsky’s differential susceptibility hypothesis [54] which suggests that some individuals might be more susceptible to negative as well as positive environmental influences in a for-better-and-for-worse-manner than others. Future studies should examine the function of this altered interaction behaviour by assessing physiological measures besides interactive measures in challenging mother-infant interaction (e.g. respiratory sinus arrhythmia, [89].

#### Effects of infant sex

There was no significant main effect of infant sex but significant two-way (episode x infant sex) and three-way (group x episode x infant sex) interaction terms. All in all, post-hoc tests suggested that the different pattern of positive engagement between the clinical and control group especially referred to male infants: boys of the clinical group started with lower rates of positive engagement than control males and only these male control infants demonstrated significantly less positive interaction behaviour in the still-face episode. The girls of both groups demonstrated no significant change or group difference of positive interaction in the course of the FFSF. This outcome and the finding that males generally showed more positive engagement during the play phase than females may suggest a higher social responsiveness as well as a higher vulnerability to maternal anxiety symptoms for male infants. In line with the ideas of Weinberg and colleagues [51], boys may have less pronounced affect self-regulation skills than girls at this age, thus, rely greatly on their caregivers’ regulation. The increased social responsiveness to maternal interaction in male infants may simultaneously represent a weakness and a strength: on the one hand, male infants might be more vulnerable to adverse effects of negative experiences than female infants which is in line with the diathesis-stress model [90]. On the other hand, aligned with Belsky’s differential susceptibility hypothesis [54], male infants could be more susceptible to negative as well as to positive environmental influences in a for-better-and-for-worse-manner [54]. In other words, male infants might be affected by maternal mental health problems but also by positive conditions to a greater extent than female infants. However, female infants might be better at protecting themselves against negative interactive experiences with anxious or otherwise impaired mothers by reducing their emotional sensitivity.

Aside from infant sex specific mechanisms of emotional regulation, male infants might be more explicit when expressing their positive emotional states, reflected by the higher level of expressed positive affect. This finding is consistent with previous research showing that male infants were more likely to look at their mother, display facial expressions of joy and use more neutral or positive vocalisations in the FFSF paradigm than females [51,21]. In contrast to the female infants who looked more at objects and showed more facial expression of interest when interacting with their mother [51,91].

The higher positive engagement of boys could also reflect an infant sex specific response to female caregivers. Male infants might be more susceptible to less beneficial interaction styles of mothers than females. This interpretation is in line with prior work where emotional negativity, criticism and lack of interest of fathers during infanthood correlated with internalized behavioural problems in eight-year-old girls but not in boys [92]. Future research considering gender-specific effects within caregiver-infant dyads is needed to validate this assumption [93,2]. Braungart-Rieker and colleagues [94] found gender differences in expressed affect and emotion regulation during mother-infant but not during father-infant still-face situations. Infant interactive behaviour should, therefore, be examined in mother-infant as well as father-infant dyads to avoid overgeneralising and misinterpreting gender differences.

### Infant negative interactive behaviour

The infants of the healthy women responded promptly with increased protest to the maternal withdrawal in the still-face episode. The clinical group exhibited a significant increase in protest between the play and the reunion episode. The pronounced protest in the reunion phase in the infants of the anxious women could be linked to complex and less obvious interaction patterns [44] and affect contagion [95] through increased maternal distress [96] in anxious mother-infant dyads. This may interfere with efficient self- and dyadic-regulatory processes when resuming interaction.

Such interaction pattern of infants of mothers with anxiety disorder could be viewed in the light of reduced emotional sensitivity which possibly protects the infant against negative interactive experiences with an anxious caregiver. These notions clearly require further examination in future studies combining multimodal measures of maternal interaction behaviour and physiological arousal.

### Maternal interaction behaviour

We neither found deficits in positive engagement nor pronounced intrusiveness in mothers with anxiety disorder. Overall, maternal positive interaction and vocalisations were the most frequent engagement codes (80% for the clinical and 82% for the control group; play and reunion phase). This outcome is consistent with Kaitz et al. [64] and Weinberg et al. [21] who did not find a low degree of positive engagement or any significant differences in expressed positive affect between mothers with anxiety disorder and controls. Thus, mothers suffering from anxiety disorder may indeed possess pronounced positive interaction skills when interacting with their infant in a context such as the FFSF paradigm. As already mentioned, within the same sample positive maternal interaction behaviour moderated the relationship between maternal anxiety and infant regulatory problems [63]. Additional studies involving other samples are surely required, yet, it is possible that not anxiety disorder itself but the combination with less expressed positivity may account for less optimal regulation skills in infants.

The clear strength of the presented data is the strict exclusion of mothers with comorbid depression. Even though this might not resemble the high comorbidity of both disorders, an effect of an underlying depression can be ruled out. Two research groups, who investigated the interaction of anxious mothers with no comorbid psychological disorder, also could not find any differences between anxious and control mothers [64,21]. It is possible that depression has more detrimental effects on mother-infant interaction than anxiety disorders; a notion supported by Feldman et al. [18]: anxious mother-infant dyads performed less well in maternal sensitivity and infant social engagement than control dyads but were more sensitive and socially engaged than depressed dyads. It is important to mention that subclinical depressive symptoms and anxious avoidance, assessed by self-report questionnaires, were not related to mother-infant interaction in the given sample. Nevertheless, differences in maternal care between mothers with an anxiety disorder and controls may exist, yet, we might not have been able to detect them by using the FFSF and the microanalytical coding system ICEP-R. Future studies should use different coding systems including macro-temporal coding systems, in different observational situations (e.g. home observation vs. laboratory observation) to answer this question.

### Maternal intrusiveness and infant protest

In explorative analyses, we tested whether maternal intrusiveness and infant protest are related. According to the obtained results, maternal intrusiveness was positively associated with infant protest during free play. In addition, infant protest in the still-face episode correlated positively with maternal intrusiveness in the reunion phase. These findings imply that maternal intrusiveness is connected to dysfunctional regulation processes and distress in infants and vice versa. It is possible that children of intrusive mothers are more distressed. However, the direction of the found connection needs to be examined further.

### Conclusions and clinical implications

Our findings, if replicable, suggest that mother-infant intervention in the field of postpartum anxiety disorders should focus on infant and dyadic abilities to regulate infant affect. As previous studies underlined the significance of maternal as well as infant positive expressed emotion for infant cognitive and socio-emotional development (e.g. [9,97,98,]), interventions should especially strengthen the interactive exchange of positive emotion. It might further be fruitful to consider the increased social responsiveness of male infants to early interactional experiences with caregivers as it could simultaneously represent a strength and a weakness as formulated in Belsky’s differential susceptibility model [54]. Male infants might benefit more from interactive therapy aiming at more beneficial maternal interactive styles because of their pronounced social responsiveness. A video-based approach has become increasingly popular (e.g. [99,100]) as it allows visualising the implicit, non-verbal as well as verbal interactive processes of the mother-infant dyad.

It is of great importance to address the influence of maternal anxiety disorder in longitudinal studies to improve the knowledge of possible developmental and interactive consequences. Specific intervention strategies could then enhance maternal dyadic regulatory competencies to prevent regulatory problems in infancy and childhood. Future studies should also reinvestigate infant protest with more comprehensive and homogeneous samples including physiological data.

### Limitations

Although typical for many clinical studies, our sample size was relatively small. Moreover, the sample consisted largely of participants with a university degree; limiting the generalisability of our results. Our sample is characterised by a wide infant age range due to recruitment difficulties. We tried to statistically control for infant age by adjusting ANOVAs to infant age when necessary but error cannot definitely be partialled out completely by the means of mathematics. Future studies should rely on homogeneous age samples when examining the impact of postpartum anxiety disorder on mother-infant interaction.

More than half of the clinical sample suffered from more than one anxiety disorder. Research suggests that the influence of maternal anxiety disorder on mother-infant behaviour varies with the sub-type of anxiety disorder [101,102]. Because of the high comorbidity of anxiety disorder in the presented sample, it was not possible to test for such specific associations. However, multiple anxiety disorders are rather the norm than the exception in clinical practise. In addition, comorbid anxiety disorders have been related to higher chronicity, treatment and severity [66]. Our sample might thus represent a highly impaired sample that allows testing for possible effects of anxiety disorders on mother-infant interaction.

It is possible that the low risk characteristics of the given sample (e.g. high educational degrees and stable relationships) account for the higher degree of positive interaction. Less pronounced interactive difficulties have been found in low risk samples of depressed mothers, while socially disadvantaged depressed mothers demonstrated greater disturbances in mother-infant interaction [103,104,105]. Future studies involving high risk samples would clarify this assumed buffering effect.

A power analysis (Table 1) showed that neither large effects in all analyses nor medium-sized effects for the repeated-measures effects can be ruled out in case of null-findings. Small effects cannot be ruled out as well as medium-sized effects for the between-subject effects. However, especially post-hoc tests might have ran out of power since cell frequencies are limited to the subjects constituting the means of the effect of interest. Consequently, regarding a validation of null-findings and the two-way and three-way interaction effects, larger sample sizes are needed. Due to cross-sectional data acquisition, we cannot draw any causal conclusions.

## Supporting information

S1 File Infant and Caregiver Engagement Phases (ICEP)—Heidelberg Version. (PDF)

S2 File Minimal anonymized dataset. (XLSX)

S3 File Dataset description. (DOCX)
