# Supplementary material for: The impact of maternal anxiety disorder on mother-infant interaction in the postpartum period
Source: PLoS One. 2018 May 25;13(5):e0194763. doi: 10.1371/journal.pone.0194763 (PMC5969737; doi:10.1371/journal.pone.0194763)
Supplement: S1 File — (PDF) [file pone.0194763.s001.pdf]

Infant and Caregiver Engagement Phases (ICEP)

Heidelberg Version

Revised Version 08/2009:
Corinna Reck, Daniela Noe & Francesca Cenciotti
Department of General Psychiatry, University of Heidelberg, Germany

Original Version 07/1999:
M. Katherine Weinberg & Edward Z. Tronick
Children's Hospital and Harvard Medical School, Boston, MA

The Infant and Caregiver Engagement Phases are based on Tronick's Monadic Phases Scoring System, Tronick and Weinberg's Infant and Maternal Regulatory Scoring Systems (IRSS & MRSS), and Weinberg and Tronick's work on affective configurations (Child Development, 65(5)). This coding system can be used to separately assess infant and caregiver affect, the extent to which the infant and caregiver are engaged, as well as the quality of the engagement. The codes within the Infant and Caregiver Engagement Phases are mutually exclusive and may be coded using interactive coding software, a VCR computer interface system or paper and pencil. Code in separate runs the infant and caregiver phases, the additional infant and caregiver codes and the dyadic gaze information.

Coding Basics:

- Always refer to the manual throughout coding.
- Watch the segment to be coded in its entirety before coding.
- Remember, BEHAVIOR is being coded not what the infant appears to be feeling.
- Have another coder look at the segment if you are unsure of the appropriate code.
- Do not code for long periods without a break. If you are tired and find yourself having a more difficult time than usual, please stop and come back to coding later.
- Don't forget the step-down rules. If you have to watch a segment more than twice, step down to the more neutral code.

Coding Process:

1. infant engagement phases
2. caregiver engagement phases
3. additional codes infant
4. additional codes mother
5. dyadic gaze information

INFANT ENGAGEMENT PHASES

The infant engagement phases are mutually exclusive. The phases combine information from the infant's facial expressions, direction of gaze, body posture and vocalizations.

1. **Negative Engagement (Ineg):** The infant is negative, protesting or withdrawn. The infant must display negative facial expressions (e.g., anger, sadness, disgust, distress, cry or grimace faces), and/or whimpering, complaining, fussy, or crying vocalizations. There is no gaze criterion and a variety of gestural and postural behaviors may occur (e.g., pushing the caregiver away, twisting and turning in chair, arching body).

Negative Engagement can be divided into two specific phases: Protest and Withdrawn. Always try to code whether the infant is in a Protest or Withdrawn phase. If it is not possible to make this differentiation, code the undifferentiated category of Negative Engagement. The overall feel for this code is unhappy or sad without the further differentiation of the Protest or Withdrawn codes [In typical populations, Protest will be the most common negative code and Withdrawn will be rare].

2. **Protest (Ipro):** The infant is protesting. The infant often displays facial expressions of anger, frustration, grimaces, and/or is fussing or crying. The infant tends to be active during this phase: the infant may arch his/her back, kick or bang his/her arms against the chair, try to escape /get away, gesture, want to be picked up, bat at the caregiver, or push and pull away from the caregiver. This code captures externalizing type behaviors, and can have an angry or hostile aspect. There is no gaze criterion.

3. **Withdrawn (Iwit):** The infant is withdrawn and minimally engaged with the caregiver. This phase often includes sad facial expressions, whimpering vocalizations, slumped posture, listless demeanor, and gaze aversion. The infant typically engages in few activities and gives the impression of being "spaced out" and disengaged from the caregiver. This code captures internalizing type behaviors. Although the infant often gaze averts, there is no specific gaze criterion defining this phase. Do not code withdrawn if the infant is crying or looking away from the caregiver but is focused on an object.

4. **Object/Environment Engagement (Inon):** The infant is looking at objects that are either proximal (e.g., infant seat) or distal (e.g., camera). The infant may manipulate proximal objects. The infant's eyes must be directed towards an object. The infant's facial expressions are typically interested or neutral but may be on occasion positive. The infant may or may not vocalize. Objects include the infant's hands, feet, belly or clothing; the caregiver's body (e.g., trunk, hands, jewelry); and objects that are part of the laboratory setting (e.g., chair strap, side of the chair, cameras or curtains). The caregiver's face does not constitute an object. If the infant displays negative affect or vocalizations while looking at an object, code Negative Engagement, Protest or Withdrawn. If the infant laughs or smiles while looking at an object, code Object/Environment Engagement not Social Positive Engagement (ipos).

5. **Social Monitor (Ineu):** The infant's attention is directed towards the caregiver. The infant looks at the adult's face with a neutral or interested facial expression. The infant's eyes must be oriented towards the adult's face and he/she may vocalize in a neutral/positive manner. In cases where it is difficult to differentiate between Negative

Engagement (particularly withdrawn) and Social Monitor, score Social Monitor. Similarly, if it is difficult to differentiate between Social Monitor and Social Positive Engagement, score Social Monitor. If it is difficult to differentiate between Object Engagement and Social Monitor, score Object Engagement.

6. **Social Positive Engagement (Ipos):** This scale assesses the extent to which the infant is engaged with the caregiver in an overall positive manner. The infant must display facial expressions of joy particularly smiles, but occasionally coo and play faces. The infant must look towards the caregiver's face. The infant may be vocalizing in a positive manner, laughing, babbling, or squealing. Do not code Social Positive Engagement if the infant vocalizes but does not display a smile face. A smile face includes upturned mouth, crinkly eyes and raised cheeks. The infant may be engaged with the adult in rhythmic social play-games (e.g., pat-a-cake, peek-a-boo). During these games it may not be possible for the infant to look directly at the adult's face because of the caregiver's position (e.g., adult's face buried in infant's lap) or the nature of the game (e.g., face covered in peek-a-boo). Score these instances as Social Positive Engagement as long as the infant's facial and/or vocal expressions are positive and gaze is focused towards the caregiver.

7. **Sleep (Islp):** The infant is asleep.

8. **Unscorable (Iusc):** If the baby's face is obscured because of poor camera angles, technical problems, or because the adult is blocking the baby's camera, score Unscorable. If it is possible to see part of the infant's face, code the appropriate engagement phase. Do not guess. In general, use the following rules to code Unscorable. Even though the infant's face is blocked, if it is clear that the infant is looking away from the mother, score Object/Environment Engagement. If the infant's face is obscured and the infant is fussing or crying, score the appropriate Negative Engagement code. If the infant's face is obscured and the infant seems to be looking in the direction of the mother, score Unscorable because it will be impossible to know exactly where the infant is looking or to determine the infant's facial expression. However, if the infant does not move and is in the same coding behavior from the beginning of being obscured to being observable then score that coding behavior.

A note on the use of pacifiers: The use of pacifiers is problematic because they obscure the baby's mouth. Use the following rules if a pacifier is in use: Code Social/Positive Engagement if it is CLEAR that the baby is smiling (e.g., the cheeks are clearly raised even with the pacifier). If this is not clear, use the default rule and code Social Monitor (but only if the baby is looking at the parent). If the baby is crying/fussing with a pacifier in the mouth, code the appropriate Negative Engagement code. If the baby is looking away from the caregiver and is not crying/fussing, score Object/Environment Engagement. If the baby is looking away and fussing/crying, code the appropriate Negative Engagement code.

A note on SNEEZING: If the infant sneezes and involuntarily closes his/her eyes, append the sneezing fit to the previous code. For example, if the infant looks at an object, sneezes, and then smiles at the mother, code a 4 and a 6. If the infant looks at an object, sneezes and continues to look at an object, code a continuous 4.

CAREGIVER ENGAGEMENT PHASES

The Caregiver Engagement Phases are mutually exclusive. For each phase, consider the adult's facial expressions, direction of gaze, and vocalizations.

1. **Negative Engagement (Cneg):** The adult is negative, withdrawn, intrusive or hostile. His/her facial expressions are sad, sober, expressionless, stern, angry or hostile. There are no smiles or hints of smiles. The adult's vocalizations are expressionless, sharp, angry, loud or adultlike. The adult may be silent or speak in a monotone. The adult may be leaning back in his/her chair and appear at a loss of what to do. There is no burst-pause, sing-song, or exaggerated language characteristic of motherese. When considering the adult's vocalizations, pay attention to the affective tone of the vocalizations and disregard the content of the vocalizations. There is no gaze criterion.

According to the main affect negative Engagement can be divided into three specific phases: Withdrawn, Intrusive and Hostile. Always try to code whether the adult is in the Withdrawn, Intrusive or Hostile phase. If it is not possible to make this differentiation, code the undifferentiated category of Negative Engagement. If Intrusive and Hostile behavior can be observed at once, code Hostile.

2. **Withdrawn (Cwit):** The adult is minimally engaged and withdrawn with the baby. The adult's facial expressions are sad, flat, or expressionless. There are no smiles or hints of smiles. The adult may be silent, speak or whisper in a flat or expressionless monotone. The adult may be leaning back in his/her chair, not touch the baby or touch it in a mechanical manner, and appear hesitant or at a loss of what to do. There is no burst pause, sing-song, or exaggerated vocalizations (i.e., motherese). There is no gaze criterion.

3. **Intrusive (Cint**, main affect „Tension /Agitation", videos: 43, A1-076): The adult's engagement with the baby is characterized by intrusive affect/behavior. The adult's facial expressions are tense, stressed or little authentic. The infant is not given the chance to respond to maternal initiatives as these follow one another too fast and do not await infant reactions. Maternal and infant reactions are not well-matched so that for example the mother interrupts the infant's activities in order to pursue her own „programme". The mother stimulates the infant excessively without caring about infant signals of discomfort and/or withdrawal. Regardless of the infant's behavior the mother acts too loud, too expressively and too close to her child. The infant's activities are restrained, for example by turning his or her face around in order to make eye contact, by holding tight hands or feet while playing etc.

4. **Hostile (Chos**, main affect „Anger/Hostility/Petulance ", videos: 21, 48): The adult's engagement with the baby is characterized by hostile affect/behavior. The adult's facial expressions are stressed out, upset or aggressive. Vocalizations are high pitched or the voice is cracking. The mother is curt with the baby, pushes him/her, drags or pulls him/her or her actions and vocalizations follow one another staccato-like. She makes fun of her baby, for example by imitating his/her vocalizations or mimic expressions.

5. **Non-Infant Focused Engagement (Cnon):** The adult is not attending to the baby and is involved in a non-infant focused activity (e.g., fixing his/her clothing, talking to the experimenter, looking at an object the infant is not looking at, rubbing eyes and face because of tiredness).

5. **Social Monitor/No Vocs or Neutral Vocs (Cneu):** The adult watches or focuses his/her attention on the baby or the baby's activities while his/her facial expressions are neutral. The caregiver may look interested in the infant and may occasionally show a hint of a smile. The adult may touch the baby. The adult can be silent or vocalize to the baby in a neutral manner. Do not code brief conversational pauses (no vocs with neutral face) as 6 unless these pauses last 2 seconds or more. Comforting sounds like shhh are coded as 6. If the caregiver's face is neutral but he/she is speaking in Motherese or in a positive tone of voice, score Phase 7 Social Monitor/Positive Vocs. In cases where it is difficult to differentiate between the Negative Engagement codes (particularly Withdrawn) and Social Monitor/No Vocs or Neutral Vocs, score a 6. Similarly if it is difficult to differentiate between Social Monitor/Neutral and Social Monitor/Positive Vocs, score Social Monitor/No Vocs or Neutral Vocs.

6. **Social Monitor/Positive Vocs (Cpvc):** The caregiver's gaze is focused on the infant or on the infant's activities. His/her facial expressions are neutral, interested, and may occasionally show a hint of a smile. Although the caregiver's face is neutral, his/her vocalizations are positive (e.g., he/she may use Motherese, make kissing or clicking sounds, sing). In cases where it is difficult to differentiate between Social Monitor/Positive Vocs and Social Positive Engagement, score Social Monitor/Positive Vocs.

7. **Social Positive Engagement (Cpos):** The caregiver expresses positive affect such as full smiles (closed or open), laughter, or play faces. The adult may vocalize to the baby using Motherese or sing but there is nothing exaggerated in his/her speaking or singing. The caregiver may play games with the infant but these games do not have a neutral quality (code 6 or 7). Instances when vocalizations (e.g., "boop") make it physically impossible to smile are coded as 8 if they are preceded by an 8. If the "boop" is preceded by a neutral face, code a 7. If, after the vocalization, the caregiver's face is not positive (e.g., neutral), code the appropriate code. Similarly, if the caregiver covers his/her face or hides his/her face in the infant's body, code these instances as 8 if they are preceded by an 8. If the caregiver emerges with a non-positive face (e.g., neutral or negative), code the appropriate code.

8. **Unscorable (Cusc):** If the adult's face is obscured because of poor camera angles or technical problems score Unscorable. Code all instances, however brief if the caregiver's face is completely obscured and if it is not possible to hear the caregiver's vocalizations. If it is possible to see part of the caregiver's face, code the appropriate engagement phase. Also, if you cannot see the face but can hear the caregiver talk using neutral or positive vocalizations, code 6 or 7. Do not guess.

ADDITIONAL INFANT AND CAREGIVER CODES

In a separate run from the phases, code infant self-comforting, distancing, and autonomic stress indicators. These infant additional codes are not mutually exclusive. Thus an infant can self-comfort while distancing him/herself from the caregiver. During this run, also code maternal rough touches and still-face violations.

If using the AACT system, code the beginning and end times of each episode during this run. Make sure that the same begin and end times are used for the infant and the caregiver. When code the appropriate episode code and the tran codes demarcating the transitional 15 sec periods between episodes.

INFANT

Self-comforting (sc)

Oral self-comforting and self-clasp may co-occur or occur sequentially. Score all instances of each.

1. **Oral Self-Comforting (Isc o):** The infant uses his/her body to provide self-stimulation. Self-comforting activities include: (1) instances when the infant sucks on his/her body (e.g., his/her thumb or wrist). There must be skin contact with the mouth and the behavior must be initiated by the infant; (2) instances when the infant sucks on or brings to his/her mouth something other than his/her body such as the strap of the chair or his/her clothing. This behavior must also be initiated by the infant; and (3) instances when the infant sucks on or brings to his/her mouth the mother's hand or finger. There must be skin contact but this self-comforting behavior is scored regardless of who initiated the contact.

2. **Self-Clasp (Isc h):** The infant's two hands are touching. Score if the hands are clearly clasped together. Score also when the hands/fingers are only lightly touching. Do not score if the place of contact is on the wrists or arms. Only hand-to-hand contact is scored as a self-clasp.

Distancing (Idis): The infant attempts to increase his/her physical distance from the caregiver without engaging an object. Distancing includes: (1) instances when the infant tries to get away by turning and twisting away from the caregiver. The infant's shoulders and trunk are rotated sideways (the shoulders and trunk need not be completely rotated but some rotation must be evident) and the infant's head is averted sideways, or up and away from the adult. The arms are usually, but not always raised above or at the level of his/her head. The back is typically, but not always arched. Do not score infants who have this constellation of behaviors but are trying to get a better look at an object; and (2) instances when the infant's shoulders are pushed back against the chair and the torso is thrust forward and up. There is no shoulder or trunk rotation. The infant's arms are usually down by the infant's sides but are occasionally raised. The infant typically looks at the adult but head and gaze are sometimes averted.

Infant Autonomic Stress Indicators (Iaut): The infant exhibits behaviors, which may indicate stress or autonomic arousal such as spitting up or hiccupping. Drooling should not be coded as spitting up. The infant's spit up should have consistency and be white or milky.

CAREGIVER

Rough Touches (tch)

Code only touches that are sharp and abrupt in nature. Ignore all other touches.

(Ctch): The caregiver's touches are abrupt, forceful, and sharp. Examples include pokes and jabs to the infant's face or head that result in the infant's head moving. Code all instances of rough touches separately even if they occur in rapid succession.

Violations (Cxst): This code is typically used during the still-face. During the still-face, the code indicates that the caregiver violated the still-face by touching or talking to the baby. Violations that involve facial expressions are not coded as Cxst but are coded with the caregiver engagement phases (If there is no violation, phase 5 will be coded).
Also code Cxst, if the mother violates the protocol during any episode by for example using a pacifier, bottle or toy. Keep in mind that only the introduction of objects not inherent to the face-to-face setting constitutes a violation. Thus, if the baby is wearing a bib and the mother plays with it, if the mother takes the baby's shoe off and plays with it, or if the mother takes her watch off and uses it as an object/toy, these instances are not considered violations. These objects are all inherent to the setting or part of the baby's/mother's clothing. The mother is being creative but is not directly violating the rules of the face-to-face procedure. However, mothers are explicitly told not to use bottles, pacifiers, and toys during the paradigm. The introduction of any of these items should therefore be coded as a violation of the still-face rules.

DYADIC GAZE INFORMATION

Dyadic Eye-Contact (dec): infant and caregiver are coordinating their gaze by looking at each other’s face

Joint Activity/Looking (jal): infant and caregiver are jointly attending to the same object. The infant and the mother are coordinating their gaze but are not looking at each other. The infant must be looking at the object; while the caregiver’s gaze may shift from infant to object. Objects may be the caregiver’s hand (e.g. butterfly hands, Itsy Bitsy Spider), a toy held by the mother, or an infant body part (e.g. they are both attending to the infants foot because the mother is playing “Piggy goes to market”) and the game is not a facial interplay game (e.g., peek-a-boo), but one in which the hands are equivalent to an object. While this code requires a mutual interaction, it captures the infant’s perspective of a social interaction with the caregiver via an object. The infant can display any facial expression.
