# Supplementary material for: The impact of maternal anxiety disorder on mother-infant interaction in the postpartum period
Source: PLoS One. 2018 May 25;13(5):e0194763. doi: 10.1371/journal.pone.0194763 (PMC5969737; doi:10.1371/journal.pone.0194763)
Supplement: S3 File — (DOCX) [file pone.0194763.s003.docx]

**The impact of maternal anxiety disorder on mother-infant interaction in the postpartum period**

Corinna Reck^1*^, Alexandra Tietz^2^, Mitho Müller^1^, Kirsten Seibold^2^, Edward Tronick^3, 4^

^1^Ludwig-Maximilians University, Department of Psychology, Munich, Germany

^2^ Heidelberg University Hospital, General Psychiatry, Heidelberg, Germany

^3^University of Massachusetts, Boston, United States of America

^4^Harvard Medical School, Boston, United States of America

*Corresponding author

E-mail: [Corinna.Reck@psy.lmu.de](mailto:Corinna.Reck@psy.lmu.de) (CR)

**Study Description**

The sample stems from a comprehensive longitudinal study dealing with postpartum anxiety disorder, mother-infant interaction and infant development. The presented data refer to the first assessment at an average infant age of four months (mother-infant interaction and diagnostic assessment). Subjects were recruited between June 2006 and October 2010 in a German town by means of flyers, newspaper advertisements and public birth announcements. Women were also recruited from the General Psychiatry or the University Women’s Hospital. Initially, the sample comprised *N* = 122 women. Study approval was obtained from the Medical Ethics Committee of the University. Written informed consent was obtained.

A full version of the German Structured Clinical Interview for DSM-IV Disorders (SCID-I) was carried out for a classification as a healthy or anxious mother. Women of the clinical sample had to fulfil the criteria for a DSM-IV diagnosis of anxiety disorder. Women with a lifetime diagnosis of psychosis and bipolar disorder were not included. Despite initial screening efforts to exclude mothers with any comorbid psychological disorder, *n* = 3 women of the clinical group were diagnosed with comorbid major depression at the first assessment. These cases were excluded. Healthy controls should neither have a current nor lifetime SCID-I diagnosis and nor have received psychotherapy at any time of their life. A total of *N* = 122 women was approached. *n* = 14 women did not meet diagnostic criteria after the first assessment (including the *n* = 3 mothers with comorbid depression) and further *n* = 16 women were recruited at a later study entry (after the video assessment). Consequently, *N* = 89 dyads were assessed for the relevant study variables. Of this sample the video recordings of *n* = 2 dyads were missing due to technical reasons. All infants were healthy and had a gestational age at birth of no less than 37 weeks.

Mother-infant interaction was investigated in a video laboratory with Tronick’s Face-to-Face-Still-Face paradigm, consisting of three episodes, each of two minutes duration, namely the play, the still-face and the reunion episode. The interactive behaviour of infant and mother during the FFSF was coded using the German translation and revision of the micro-analytical Infant and Caregiver Engagement Phases (ICEP-R).

**Sample Description**

To assure that this procedure of list-wise case-exclusions was valid for our data set, we used Little’s MCAR-test. For the MCAR-test, we considered the following variables: maternal age and education, marital status, number of children, infant age and sex, birth mode, gestation age, APGAR values, diagnostic group and interaction data (ICEP-R). The test turned out to be non-significant (χ² = 134.001, *df* = 218, *p* > .999). The final sample (*N* = 87) is comprised of *n* = 39 Caucasian women with postpartum anxiety disorder and *n* = 48 Caucasian healthy women without a history of mental health disorders, each with their infant. In the clinical sample, *n* = 24 women suffered from more than one DSM-IV anxiety disorder. *n* = 21 women had a generalized anxiety disorder; *n* = 17 women were diagnosed with panic disorder with or without agoraphobia or agoraphobia without history of panic disorder; *n* = 17 women had an obsessive-compulsive disorder; *n* = 9 women were diagnosed with social phobia; *n* = 1 woman suffered from post-traumatic stress disorder; *n* = 4 women were diagnosed with an anxiety disorder not otherwise specified. According to a retrospective self-report of the participants, *n* = 7 women had a postpartum onset of anxiety disorder. All women were symptomatic at the time of assessment.

Within the overall sample, the age of women ranged from 22 to 45 years with a mean age of *M* = 33.0 years (*SD* = 5.6 years). Infants had a mean age of *M* = 4.1 months (*SD* = 1.5 months). With regard to the total sample, *n* = 54 (62.1 %) of the infants were female. The distribution of maternal education in the overall sample was as follows: four mothers (4.6 %) had low level secondary education, *n* = 16 (18.4 %) completed intermediate secondary education, *n* = 19 (21.8 %) qualified for university entrance and *n* = 48 (55.2 %) of the mothers held a university degree. More than half of the sample had one child including the index infant (*n* = 54, 62.1 %). *n* = 26 mothers (29.9 %) had two children. *n* = 7 (8.0 %) study infants had two or more siblings. Two thirds of the sample was married (*n* = 58, 66.7 %). Overall, the sample is comparable to a representative study sample for this particular region in Southern Germany.

There was no effect of group or infant sex on maternal age, infant age, maternal education or marital status. Moreover, sex ratio did not differ between infants of the clinical and the control group. Only number of children differed significantly with clinical mothers having fewer children than controls. Descriptive data of subgroups and tests on comparability are presented in Table 1.

| **Table 1. Descriptives of subgroups and tests on comparability.** | | | | |
| --- | --- | --- | --- | --- |
|  | control group | clinical group | female infants | male infants |
| *maternal age (years) M* (*SD*) | 33.5 (5.6) | 32.3 (5.6) | 33.1 (5.4) | 32.8 (6.0) |
| *t* (*p*) | 0.98 | (.33) | 0.18 | (.86) |
| *infant age (months) M* (*SD*) | 3.9 (1.4) | 4.4 (1.5) | 4.3 (1.6) | 3.9 (1.3) |
| *t* (*p*) | 1.76 | (.08) | 1.21 | (.23) |
| maternal education | control group (*f*) | clinical group (*f*) | female infants (*f*) | male infants (*f*) |
| university degree | 28 | 20 | 30 | 18 |
| university entrance qualification | 11 | 8 | 10 | 9 |
| high secondary qualification | 8 | 8 | 12 | 4 |
| low secondary qualification | 1 | 3 | 2 | 2 |
| *U* (*p*) | 837.5 | (.35) | 875.0 | (.88) |
| one child | 24 | 30 | 38 | 16 |
| two children | 18 | 8 | 12 | 14 |
| three or more children | 6 | 1 | 4 | 3 |
| *U* (*p*) | 668.5 | (< .01) | 707.5 | (.06) |
| not married | 10 | 14 | 15 | 9 |
| married | 35 | 23 | 36 | 22 |
| χ² (*p*) | 2.39^a^ | (.12) | < .01^b^ | (.97) |
| female infants | 31 | 23 | / | / |
| male infants | 17 | 16 | / | / |
| χ² (*p*) | 0.29^c^ | (.59) | / | / |
| ^a^0 cells have expected count of less than 5, minimum expected count is 10.83.  ^b^0 cells have expected count of less than 5, minimum expected count is 9.07.  ^c^0 cells have expected count of less than 5, minimum expected count is 14.79. | | | | |

**Study Variables**

Following guidelines (<http://www.bmj.com/content/340/bmj.c181.long>, retrieved Dec, 18^th^ 2017), we reduced the data set to avoid the risk of identifying single participants. Thus, the minimal data set contains only three sociodemographic variables and the main variables to replicate our results. Information on subject ID, subcategories and number of anxiety disorders, maternal age, maternal education, marital status, birth mode, gestation age and APGAR score have been deleted, as these variables were not relevant in the reported analyses. Furthermore, all variables not analysed in the current data set were deleted. Consequently, we expect this data set to be fully anonymised and the risk of identifying single subjects as negligible. The data set contains the following variables:

| **Variable** | **Explanation** | **Labels / range / possible range** |
| --- | --- | --- |
| diagnosis | Dummy variable: diagnostic status | 0 = control group  1 = anxiety group |
| infant_sex | Dummy variable: sex of index infant | 0 = female  1 = male |
| infant_age | Parametric variable: infant age at assessment in months | Range: 2.53 – 7.93 months |
| number_infants | Parametric variable: number of infants living in household | Range: 1 – 4 infants |
| infant_positive_play | Parametric variable: relative time duration of infant positive engagement during play episode | Possible range: 0.00 – 1.00 |
| infant_positive_still_face | Parametric variable: relative time duration of infant positive engagement during still-face episode | Possible range: 0.00 – 1.00 |
| infant_positive_reunion | Parametric variable: relative time duration of infant positive engagement during reunion episode | Possible range: 0.00 – 1.00 |
| infant_protest_play | Parametric variable: relative time duration of infant protest during play episode | Possible range: 0.00 – 1.00 |
| infant_protest_still_face | Parametric variable: relative time duration of infant protest during still-face episode | Possible range: 0.00 – 1.00 |
| infant_protest_reunion | Parametric variable: relative time duration of infant protest during reunion episode | Possible range: 0.00 – 1.00 |
| infant_monitoring_play | Parametric variable: relative time duration of infant social monitoring during play episode | Possible range: 0.00 – 1.00 |
| infant_monitoring_still_face | Parametric variable: relative time duration of infant social monitoring during still-face episode | Possible range: 0.00 – 1.00 |
| infant_monitoring_reunion | Parametric variable: relative time duration of infant social monitoring during reunion episode | Possible range: 0.00 – 1.00 |
| infant_object_play | Parametric variable: relative time duration of infant object engagement during play episode | Possible range: 0.00 – 1.00 |
| infant_object_still_face | Parametric variable: relative time duration of infant object engagement during still-face episode | Possible range: 0.00 – 1.00 |
| infant_object_reunion | Parametric variable: relative time duration of infant object engagement during reunion episode | Possible range: 0.00 – 1.00 |
| maternal_positive_play | Parametric variable: relative time duration of maternal positive engagement during play episode | Possible range: 0.00 – 1.00 |
| maternal_positive_reunion | Parametric variable: relative time duration of maternal positive engagement during reunion episode | Possible range: 0.00 – 1.00 |
| maternal_vocalisation_play | Parametric variable: relative time duration of maternal positive vocalisation during play episode | Possible range: 0.00 – 1.00 |
| maternal_vocalisation_reunion | Parametric variable: relative time duration of maternal positive vocalisation during reunion episode | Possible range: 0.00 – 1.00 |
| maternal_monitoring_play | Parametric variable: relative time duration of maternal social monitoring during play episode | Possible range: 0.00 – 1.00 |
| maternal_monitoring_reunion | Parametric variable: relative time duration of maternal social monitoring during reunion episode | Possible range: 0.00 – 1.00 |
| maternal_intrusive_play | Parametric variable: relative time duration of maternal intrusive engagement during play episode | Possible range: 0.00 – 1.00 |
| maternal_intrusive_reunion | Parametric variable: relative time duration of maternal intrusive engagement during reunion episode | Possible range: 0.00 – 1.00 |

For further assistance regarding this data set, please contact the third author, Mitho Müller ([mitho.mueller@psy.lmu.de](mailto:mitho.mueller@psy.lmu.de))
